# Applications and Mechanisms of Tripterygium *Wilfordii Hook. F.* and its Preparations in Kidney Diseases

**DOI:** 10.3389/fphar.2022.846746

**Published:** 2022-03-21

**Authors:** Xue Tong, Yanheng Qiao, Yuanjian Yang, Haizhao Liu, Zhiyong Cao, Bo Yang, Lijuan Wei, Hongtao Yang

**Affiliations:** ^1^ Department of Nephrology, First Teaching Hospital of Tianjin University of Traditional Chinese Medicine, Tianjin, China; ^2^ National Clinical Research Center for Chinese Medicine Acupuncture and Moxibustion, First Teaching Hospital of Tianjin University of Traditional Chinese Medicine, Tianjin, China; ^3^ Tianjin University of Traditional Chinese Medicine, Tianjin, China; ^4^ Tianjin Jinnan Traditional Chinese Medicine Hospital, Tianjin, China

**Keywords:** tripterygium wilfordii hook. f., tripterygium wilfordii hook. f. preparations, kidney diseases, clinical applications, mechanisms, toxicity

## Abstract

*Tripterygium wilfordii Hook. f.* (TwHF) is a Chinese botanical drug containing a large number of metabolites. The discovered and recognized anti-inflammatory and immune-regulating effects have made it attract more and more attentions in trials and clinical researches. The extraction and processing of TwHF for pharmaceuticals is a manifestation of the role of traditional Chinese medicine. However, TwHF is toxic. Optimization of TwHF preparations has become a requirement for the development of TwHF pharmaceuticals. Our article introduces the main preparations of TwHF on the Chinese market and their characteristics. In particular, we summarize the clinical applications and influential mechanisms of TwHF and its preparations in kidney diseases. Considering that nephropathy is closely related to immune inflammation and TwHF is a botanical drug with a high number of metabolites, the application of TwHF in kidney diseases may be much more complicated. By revealing the role and mechanisms of TwHF in kidney diseases, this study aims to provide more insights to basic and clinical studies about nephropathy.

## Introduction

**FIGURE 1:**
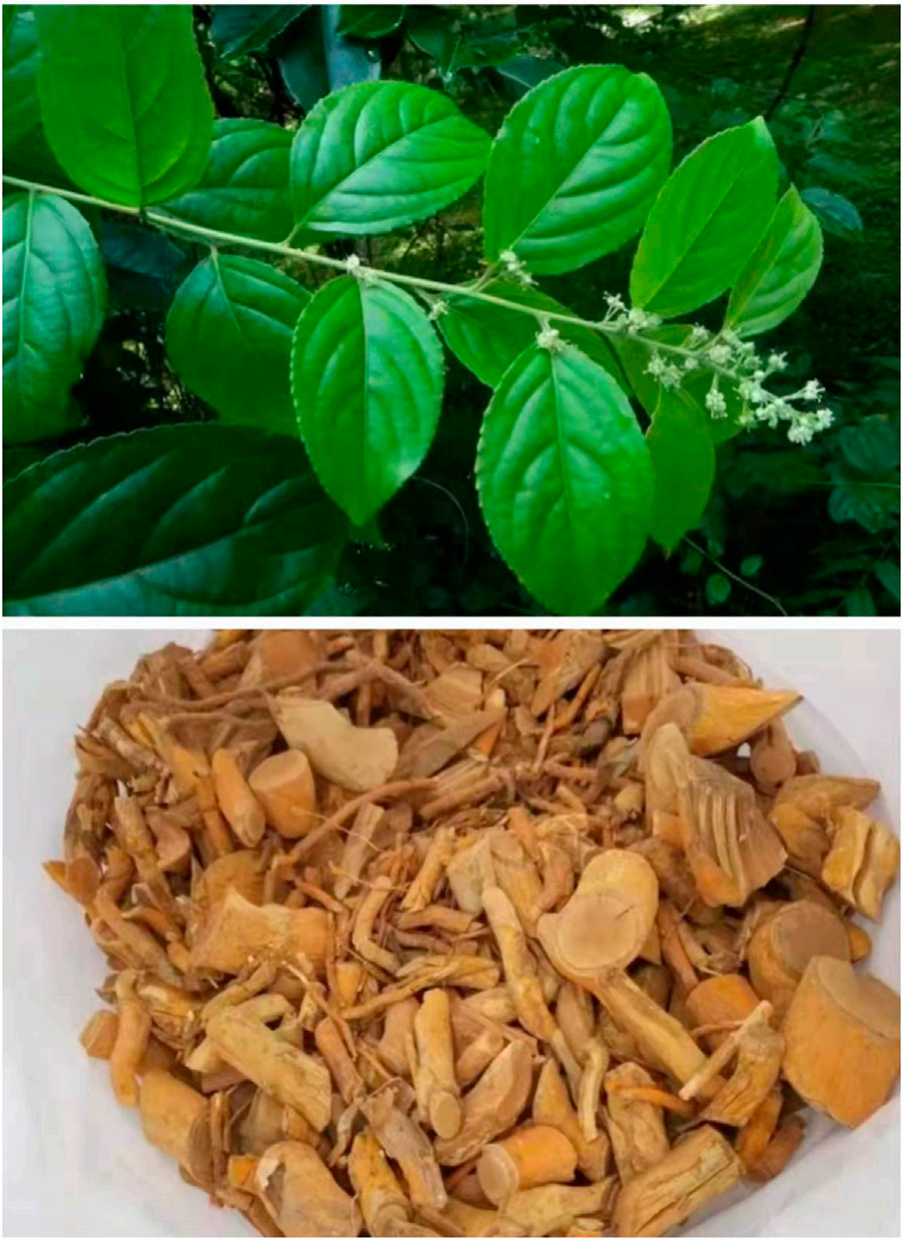
Traditional Chinese botanical drug-TwHF.

*Tripterygium wilfordii Hook. f.* (Celastraceae) (known in Chinese as Leigongteng) (TwHF) (Figure 1), the traditional botanical drug in China, was first recorded in one of the four classics of traditional Chinese medicine-the Divine Farmer’s Materia Medica and has been used in China for hundreds of years (Xiao et al., 2019). TwHF is a species of *Tripterygium Hook. f.* (Celastraceae), but the phylogenetic relationship within *Tripterygium Hook. f.* was unclear, which led to the taxonomic controversy of the genus. Previous perspectives showed that TwHF, *Tripterygium hypoglaucum (H.Lév.) Hutch.* (Celastraceae) (known in Chinese as kunmiminshanhaitang) (THH) are two species of the genus (Law et al., 2011). In recent years, based on the DNA sequencing technology, researchers have found that TwHF and THH are actually the same species (Law et al., 2011; Zhang X. et al., 2016). Si (2008) also hold the view that THH was a variant of TwHF. Several taxonomic listings [WCVP (WCVP, 2022), WFO (The World Flora Online, 2022)] have already showed that THH is a synonym of TwHF. However, the composition distribution and toxicity of the two are different (Xia et al., 1994). TwHF and THH have long been regarded as two species in traditional Chinese medicine, and various pharmacognostic methods have been developed to tell them apart (Law et al., 2011). Moreover, Chinese patent medicines that clearly use THH as ingredients have been marketed and used in China. We agree that TwHF and THH are a single species. But based on the above reasons, our review still uses the name THH to distinguish typical TwHF.

**FIGURE 2:**
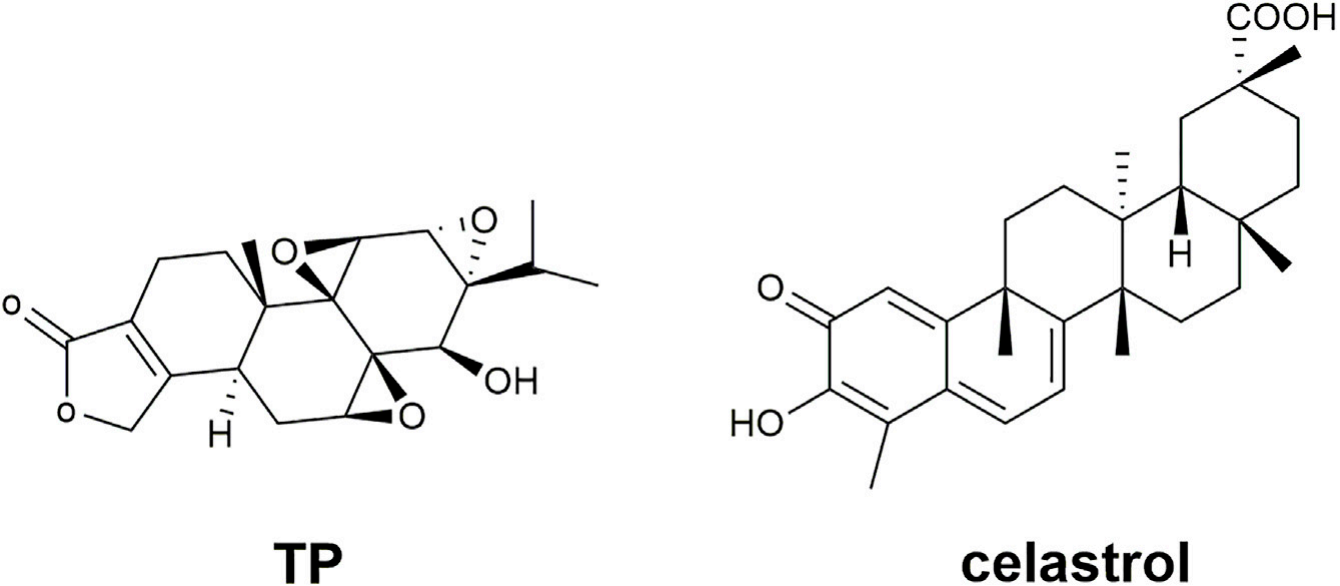
The structural formulas of TP and celastrol.

TwHF preparations include single TwHF preparations and compound preparations compatible with other Chinese medicines. TwHF itself is a botanical drug with complex and diverse chemical components. At present, there are more than 400 natural metabolites isolated and characterized from TwHF, mainly alkaloids, triterpenoids, diterpenoids and sesquiterpenes (Xiao et al., 2019). The most commonly studied natural active metabolites in TwHF are listed in Table 1, including their molecular formulas, molecular weights, toxicity, and toxic target organs. In particular, the metabolites that have anti-inflammatory and immunomodulatory effects concentrate in diterpenoids and alkaloids, which are also the main toxic ingredients in TwHF that usually cause side effects (Xue et al., 2010; Nong et al., 2019). Previous studies have shown that TwHF preparations have multiple toxicities such as gastrointestinal toxicity, reproductive toxicity, liver toxicity, nephrotoxicity, and skin toxicity (Huang et al., 2016; Nong et al., 2019; Ru et al., 2019). The study of Ru et al. (2019) showed that gastrointestinal toxicity is the most commonly observed toxicity of TwHF preparations, followed by reproductive toxicity. More importantly, the toxic dosage and the therapeutic dosage are similar, which makes the safety of TwHF more concerned (Liu LL. et al., 2019). Among the diterpenoids, triptolide (TP), a triepoxide, with its structural formula showing in Figure 2, has the strongest activity and toxicity. In addition to anti-inflammatory and immune suppression, TP is also used for anti-tumor, anti-osteoporosis and other diseases (Tong et al., 2021). In addition, many constituents of alkaloids are also believed to have the effects of anti-human immunodeficiency virus (Horiuch et al., 2006), anti-tumor (Jiang et al., 2014), analgesic (Liu L. et al., 2019), etc. Among the triterpenoid, Celastrol is currently the most extensively studied component-its structural formula displaying in Figure 2. Besides the anti-tumor effect (Lim et al., 2021), it can regulate lipid metabolism and is an effective anti-obesity agent (Feng et al., 2019). It also has the potential in central nervous system diseases’ treatment (Schiavone et al., 2021). As the chemical composition of TwHF is complex, and it contains toxic ingredients, toxin reduction and efficiency enhancement have always been the goal of TwHF preparations.

**TABLE 1:** The most commonly studied natural active metabolites in TwHF.

Classification	Representative compound	PubChem CID	Molecular formula	Molecular weight (g/mol)	Toxicity	Toxic target organs
Diterpenoids	triptolide	107,985	C_20_H_24_O_6_	360.4	highly toxic	hepatotoxicity, nephrotoxicity, reproductive toxicity, cardiotoxicity, lung toxicity, gastrointestinal toxicity, and skin irritation Cheng et al. (2021)
	tripdiolide	294,491	C_20_H_24_O_7_	376.4	toxic	reproductive toxicity Zhen et al. (1995)
	triptolidenol	3,086,461	C_20_H_24_O_7_	376.4	toxic	reproductive toxicity (Zhen et al., 1995)
	16-Hydroxytriptolide	126,556	C_20_H_24_O_7_	376.4	toxic	reproductive toxicity Zhen et al. (1995)
	triptonide	65,411	C_20_H_22_O_6_	358.4	toxic	reproductive toxicity Zhang et al. (2020)
	triptophenolide	173,273	C_20_H_24_O_3_	312.4	toxic	reproductive toxicity Qi et al. (2018)
Triterpenoids	wilforlide A	158,477	C_30_H_46_O_3_	454.7	toxic	hepatotoxicity, nephrotoxicity He et al. (2021), and reproductive toxicity Qi et al. (2018)
	wilforlide B	174,362	C_30_H_44_O_3_	452.7	Toxic Ding et al. (2021)	—
	salaspermic acid	44,593,364	C_30_H_48_O_4_	472.7	not found yet	—
	demethylzeylasteral	10,322,911	C_29_H_36_O_6_	480.6	toxic	reproductive toxicity (Xue et al., 2010)
	celastrol	122,724	C_29_H_38_O_4_	450.6	highly toxic	reproductive toxicity, cardiotoxicity, hepatotoxicity, and hematopoietic system toxicity Hou et al. (2020)
	regelin	163,808	C_31_H_48_O_4_	484.7	not found yet	—
Alkaloids	euonine	162,486	C_38_H_47_NO_18_	805.8	Toxic Dong et al. (2019)	—
	wilfortrine	73,321	C_41_H_47_NO_20_	873.8	Toxic Dong et al. (2019)	—
	wilforine	601,100	C_43_H_49_NO_18_	867.9	toxic	Hepatotoxicity Miao et al. (2019)
	wilforgine	14,108,469	C_41_H_47_NO_19_	857.8	toxic	Hepatotoxicity Miao et al. (2019)
	wilforidine	16,086,522	C_36_H_45_NO_18_	779.7	Toxic Qin (2018)	—

In recent years, research on the extraction and dosage forms of TwHF has gained more and more attentions. TwHF can be viewed as a “Chinese medicinal hormone”, with anti-inflammatory and immune regulation as its main pharmacological effects. Previous studies on TwHF preparations focused more on rheumatoid arthritis. However, there are relatively limited studies about TwHF preparations on kidney diseases. The glomerulus is a capillary globule. Circular immune complexes deposition and its own antigenic properties, such as glomerular basement membrane, can form *in situ* immune complexes, which determines that kidney diseases are closely related to immune inflammation response (Kant et al., 2021). Therefore, TwHF preparations should also have a broader application in the field of kidney diseases. Our research summarizes the development of TwHF preparations in recent years and their applications and mechanisms in nephropathy, looking forward to providing more insights to basic and clinical researches.

## TwHF Preparations

TwHF preparations include single TwHF preparations and compound TwHF preparations. Table 2 displays the most commonly used single TwHF preparations on the Chinese market. TwHF tablet and kunmingshanhaitang tablet (KMSHTT) are the representatives of single TwHF preparations. Both TwHF tablet and TwHF double-deck tablet use the ethanol-ethyl acetate extraction process, so their ingredients are similar, and the content of terpenoids is relatively high (Wang et al., 2019). Unlike TwHF tablet, TwHF double-deck tablet has two decks of drug releases, fast release and sustained release, with 30% released quickly in the stomach and 70% released slowly in the intestine. This feature reduces the side effects mainly based on gastrointestinal tract and prolongs the blood concentration (Li, 2002). TwHF total terpenoids tablet is extracted with ethanol and then treated with a low-polar solvent. This method retains the terpenoids and removes the oil that irritates to gastrointestinal tract, and also reduces the side effects after taking the medicine (Wang, 1996). KMSHTT uses the dried roots of THH as a medicine. In terms of chemical composition, the roots of typical TwHF and THH are similar (Huang et al., 2010), but there are certain differences in content distribution. Xia et al. (1994) used thin-layer chromatography to compare the components of TwHF and THH. They found that the content of diterpene lactones and celastrol in TwHF is higher than that in THH, and the content in the root bark is higher than the root core in both botanical drugs. In addition, the content of the remaining triterpenoids in TwHF is less than that in THH, and the content in the root bark is less than the root core in both botanical drugs. Moreover, the content of alkaloid is the same between TwHF and THH, and for both botanical drugs, the content in the root bark is higher than the root core (Xia et al., 1994). That is to say, TwHF, especially the root bark, is more toxic, the dosage of which should be paid higher attentions clinically. Tripterygium glycosides tablet (TGT) uses peeled root of typical TwHF/THH and maybe other species of the same genus. After procedures including alcohol extraction, chloroform or chloroform-ethanol extraction, and silica gel column chromatography, the composition of TGT is relatively simple, which consists a targeted collection of terpenes ingredients and hence reduces the toxicity and side effects (Wang et al., 2019; Sun et al., 2021). THH is also called colquhounia. Colquhounia root tablet (CRT) adopts peeled roots of THH, using water-extraction and alcohol-precipitation extract technology, which reduces the toxicity and side effects as well (Lin and Qi, 2005).

**TABLE 2:** Single TwHF preparations.

TwHF preparations	Component	Part	Content determination	Dosage form	Extract process	Specifications	Usage and dosage	National drug standard (standard number)
Oral preparations								
TwHF tablet	TwHF	Dry roots	TP is 80–120% of the labeled amount	Tablet	Ethyl acetate extraction after alcohol extraction	12μg/tablet (calculated by TP)	1–2 tablets, 2–3 times/day	WS3-B-3120–98-2015
TwHF total terpenoids tablet	TwHF	Dry roots and rhizomes	TP is 80–120% of the labeled amount	Tablet	Petroleum ether degreasing after alcohol extraction	20μg/tablet (calculated by TP)	2 tablets, 3 times/day	WS-10715(ZD-0715)-2002-2011Z
TwHF double-deck tablet	TwHF	Dry roots	Total alkaloids≥1.2mg/tablet;	Tablet	Ethyl acetate extraction after alcohol extraction	50 μg/tablet (calculated by TP)	1–2 tablets, 2 times/day. Take immediately after breakfast and dinner	WS3-755- (Z-158) -2005 (Z)
			TP should be 40–60 μg/tablet					
KMSHTT	THH	Dry roots	Total alkaloids≥1.0 mg/tablet	Tablet	Alcohol extraction	1) Film-coated tablets, weight 0.29 g/tablet, 2) Sugar-coated tablets (the core weight 0.28 g/tablet)	2 tablets, 3 times/day	Chinese pharmacopoeia (2020)
CRT	THH	Peeled dry roots	Epicatechin>0.1 mg/tablet;	Tablet	water-extraction and alcohol-precipitation	0.18 g/tablet	3–5 tablets, 3 times/day	WS-11372 (ZD-1372) -2002
			TP > 1.36 μg/tablet					
TGT	TwHF/THH*	Peeled roots	TP ≤ 10μg/tablet; wilforlide A ≥10μg/tablet	Tablet	Chloroform or chloroform-ethanol extraction after alcohol extraction, silica gel column chromatography	10 mg (calculated by tripterygium glycosides)	1–1.5 mg/kg/day, 3 times after meals, or follow the doctor’s advice	WS3-B-3350–98-2011
Hydroxytrypt-olide Tablet	TP	—	—	—	—	—	—	Clinical trial stage
External preparations								
TP ointment	TP	—	TP is 90–110% of the labeled amount	Ointment	—	10 g: 0.2 mg; 20 g: 0.4 mg	External use.	WS-10001-(HD-0293)-2002
							2–3 times/day	

Note: * represents that TGT may use other species of Tripterygium Hook. f. in addition to TwHF, and THH., Because the WS3-B-3350-98-2011 standard does not attach quality standard of the extract and there are many manufacturers of TGT, we only list the main species.

**FIGURE 3:**
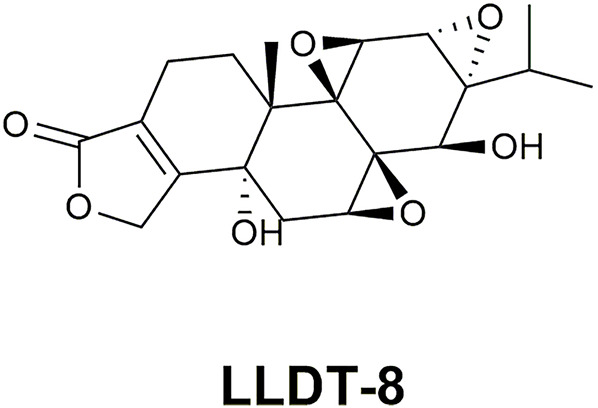
The structural formula of LLDT-8.

Hydroxytriptolide tablet, developed by shanghai institute of materia medica, consists of (5R)-5-hydroxytriptolide (LLDT-8), which is a hydroxylated derivative of TP (Xi et al., 2017) (the structural formula is shown in Figure 3). TP is the main active ingredient of TwHF, however, its strong and extensive toxicity restricts its clinical application (Zhou et al., 2005). How to maintain the biological activity while reduce the toxicity has gained increasing research popularity in recent years. LLDT-8, as a derivative of TP, remains to show effective anti-inflammatory and immunosuppressive activities (Tang and Zuo, 2012), and its toxicity is greatly reduced (Xi et al., 2017). Until now, its phase 1 + 2 clinical trials on the effectiveness and safety of rheumatoid arthritis, which is insufficient response to methotrexate, have been completed (Registration number: CTR20140491). There are also two clinical trials still in progress (Registration number: CTR20191397 and CTR20201397). LLDT-8 is expected to be the best derivative of TP.

Table 3 lists the commonly used compound TwHF preparations on the Chinese market. Gufengning capsule and Jinguan tablet are early compound TwHF preparations, which work together with other Chinese medicines that can tonify liver and kidney or promote blood circulation and dredging collaterals. There are relatively few literatures on these two drugs. Kunxian capsule (KXC) is a new generation of compound TwHF preparations, composed of THH, *Epimedium brevicornu Maxim* (Berberidaceae), *Lycium barbarum L.* (Solanaceae) and *Cuscuta chinensis Lam.* (Convolvulaceae). Each component is extracted with the macroporous resin adsorption technology, and the high performance liquid chromatography technology is adopted to detect their active ingredients, which reduce the toxicity and side effects of KXC (Lu and Yuan, 2021). A controlled experiment of KXC and TGT on the treatment of chronic kidney disease (CKD) showed that both can reduce the proteinuria level, but KXC took effects more quickly and treated more pathological types (Tu et al., 2021). Based on a Network pharmacology analysis, KXC can regulate the proliferation of inflammatory cells *via* a variety of signaling pathways. Meanwhile, Epimedium brevicornu Maxim, Lycium barbarum L., and Cuscuta chinensis Lam. can enhance the therapeutic effect of THH and weaken its side effects (Tang Y. et al., 2020).

**TABLE 3:** Compound TwHF preparations.

TwHF preparation	Component	Content determination	Dosage form	Extract process	Specifications	Usage and dosage	National drug standard (standard number)
Gufengning capsule	THH and other 10 Chinese medicines	The total alkaloid of the dried product≥ 0.93%	Capsule	Alcohol extraction and decoction	0.4 g/capsule	2–3 capsules, 3 times/day	WS-10843(ZD-0843)-2002-2012Z
Jinguan tablet	TwHF and other 17 Chinese medicines	TP should be 3.0–10.0 μg/tablet	Tablet	TwHF is washed with yellow wine; the rest is decocted with water	0.3 g/tablet	4 tablets, 3 times/day after meals	WS3-117(Z-22)-94(Z)-2009
KXC	THH and other 3 Chinese medicines	TP should be 17.5–32.5 μg/capsule; Icariin≥7.5 mg/capsule	Capsule	Decoction and macroporous resin adsorption	0.3 g/capsule	2 capsules, 3 times/day after meals. One course of treatment is 12 weeks	YBZ07522006-2009Z

## Clinical Applications and Mechanisms of TwHF and its Preparations in Kidney Diseases

### Clinical Applications

The bibliometric analysis shows that research and applications of TwHF preparations have focused on kidney diseases, rheumatic immune diseases, and immune-related skin diseases nowadays (Wang H. Y. et al., 2021). Table 4 summarizes the clinical studies of various TwHF preparations on the treatment of different kidney diseases in recent years. From the table, it can be seen that kidney diseases concerns IgA nephropathy (IgAN), membranous nephropathy (MN), chronic glomerulonephritis (CGN), nephrotic syndrome (NS), diabetic kidney disease (DKD), henoch-schonlein purpura nephritis (HSPN), lupus nephritis (LN) and chronic allograft nephropathy (CAN). The results generally show that compared with the control group, the experimental group that receives the treatments of TwHF preparations show the decline of 24-h urine total protein (24hUTP) quantity, the decline of renal function indexes such as blood urea nitrogen (BUN) and serum creatinine (Scr), the decline of blood lipid indexes such as total cholesterol (TC) and triglycerides (TG), the increase of serum albumin (ALB), the decrease of inflammatory factors [e.g., hypersensitive c-reactive protein (hs-CRP), interleukin-(IL-) 6 and tumor necrosis factor (TNF)], the up and down regulations of immune indicators [e.g., immunoglobulin (Ig) G and complement C3], as well as the lower incidence of adverse reactions and lower recurrence rate. The effective rate can be statistically significant or insignificant, suggesting that the efficacy of TwHF preparations alone or in combination with other drugs is higher than or equivalent to control group. There are also studies evaluating the effectiveness of TwHF preparations in kidney diseases using meta-analysis. For instance, Jin et al. (2021) conducted a meta-analysis of animal experiments on the treatment of DKD with TwHF, and found the adoption of TwHF in animals was also effective and safe. Chen et al. (2013) carried out a meta-analysis of TwHF preparations on the treatment of primary NS. The results showed that TwHF preparations had an additional effect on the remission of primary NS, but there was insufficient evidence to prove that they were as effective as prednisone (Pred) and cyclophosphamide (CTX). Therefrom they proposed TwHF can be directly compared with the widely used immunosuppressive agents in the future (Chen et al., 2013).

**TABLE 4:** Clinical studies of TwHF preparations on the treatment of kidney diseases.

disease	Number	Experimental group (TwHF preparation)	Control group	Background therapy	Effective rate (experimental group/control group) (%)	Experimental result (experimental group/control group) (*p* < 0.05)
IgAN	72 Wang et al. (2005)	CRT	Usual care	General therapy	91.0/67.0	Urine: 24hUTP↓, RBC count↓
						Immune: IgA↓
	34 Wang (2020)	TGT	Usual care	Olm	94.1/64.7	Urine: 24hUTP↓
						Renal function: Scr↓, BUN↓
MN	60 Xie and Dang (2016)	CRT	Usual care	Irb + General therapy	93.3/63.3	Urine: 24hUTP↓
						Serum: ALB↑
						Renal function: Scr↓, BUN↓
						Blood lipid: TC↓, TG↓
	167 Qiu et al. (2021)	TGT	Usual care	General therapy	78.7/44.3	Urine: 24hUTP↓
						Serum: ALB↑
						Others: incidence of bone marrow suppression↑
	92 Liang et al. (2020)	TGT	Usual care	FK506 + General therapy	93.5/71.3	Urine: 24hUTP↓
						Serum: ALB↑
						Immune: C5b↓,IgG4↓
						Sex hormone: SBG↑, E2↑, T↑
	55 Guo et al. (2021)	TGT	Usual care	ARB	Remission rate: 74.3/35.0	Urine: 24hUTP↓
						Serum: ALB↑
						Immune: anti-PLA2R↓
						Others: recurrence rate↓
	60 Ji et al. (2021)	KXC	FK506	Pred + General therapy	85.7/90.0	Similar effects;
						Lower incidence of adverse reactions.
CGN	84 Cui, (2016)	TGT	Usual care	SF	90.5/73.8	Urine: 24 hUTP↓
						Renal function: Scr↓, BUN↓, Ccr↑
	89 Li et al. (2021a)	TGT	Usual care	Irb/Hyd + Dip	_	Urine: 24hUTP↓
						Renal function: Scr↓, BUN↓
						Inflammatory: hs-CRP↓, TNF-α↓, IL-8↓
						Immune: CD4^+^↑, CD4^+^/CD8^+^↑, CD8^+^↓
	100 Zhao et al. (2021)	KXC	Usual care	Lef + General therapy	94.0/78.0	Urine: 24hUTP↓
						Serum: ALB↑
						Renal function: Scr↓
						Immune: IgA↑, IgG↑, IgM↑
NS	32 Kang and Cui (2011)	KMSHTT	Usual care	Pred + General therapy	_	Urine: 24hUTP↓
						Serum: ALB↑
	80 Xiong, (2019)	TGT	Usual care	Pred	90.0/75.0	Urine: 24hUTP↓
						Renal function: Scr↓, BUN↓
						Serum: ALB↑
						Others: incidence of adverse reactions↓, recurrence rate↓
	76 Chen (2018)	TGT	Usual care	General therapy	81.6/68.4	Urine: 24hUTP
						Renal function: Scr↓, BUN↓
						Inflammatory: hs-CRP↓,IL-18↓, IL-1β↓
	84 Li et al. (2020)	TGT	Usual care	Pred + CTX	92.9/73.8	Urine: 24hUTP↓
						Inflammatory: TNF-α↓,IL-6↓, hs-CRP↓
						Others: incidence of adverse reactions↓
	80 Lu (2003)	TwHF Tablet	Usual care	Dex	91.3/85.3	Higher negative conversion rate of urine protein;
						Lower recurrence rate
	87 Zhang, (2020a)	KXC	Usual care	FK506 + General therapy	98.0/80.5	Urine: 24hUTP↓
						Renal function: Scr↓, BUN↓
						Inflammatory: IFN-γ↓、IL-21↓
						Blood lipid: TC↓
						Others: incidence of adverse reactions↓
DKD	94 Zhang (2020b)	TGT	Usual care	Pred + General therapy	95.7/80.9	Inflammatory: CRP↓,TNF-β↓
	84 Xu et al. (2017)	TGT	Usual care	Tel + General therapy	81.0/52.4	Urine: 24hUTP↓
						Renal function: Scr↓, GFR↑
	102 Ma and Yao, (2020)	TGT	Usual care	Dapa + General therapy	80.4/60.8	Urine: 24hUTP↓
						Renal function: Scr↓, BUN↓
						Inflammatory: hs-CRP↓, IL-6↓, IL-8↓
						Immune: IgA↑, IgG↑, C3↑, C4↑
						Others: symptom score improved
	184 Wang et al. (2016)	CRT	Usual care	Val + General therapy	87.0/70.7	Urine: 24hUTP↓, UACR↓
						Renal function: Scr↓
						Blood lipid: TC↓, TG↓, LDL-C↓
	80 Zhou et al. (2016)	CRT	Irb	General therapy	77.5/55.0	Urine: 24hUTP↓, UACR↓
						Others: no statistically significant in incidence of adverse reactions
	60 Wang et al. (2015a)	CRT	Usual care	Irb	83.3/40.0	Urine: 24hUTP↓, UACR↓
						Renal function: Scr↓
						Blood lipid: TC↓, TG↓, HDL-C↑, LDL-C↓
						Blood pressure: SBP↓
						Others: HGF↑
HSPN	96 He et al. (2020)	TGT	Usual care	Usual care	91.7/72.9	Inflammatory: TNF↓
						Immune: CD137↓, CD137↓, IgA↓, IgG↓
	172 Zhang et al. (2021)	TGT	Usual care	FK506	_	Better long-term curative effect; lower incidence of adverse reactions;
						lower recurrence rate
LN	66 Liu et al. (2020)	KXC	CTX	Pred	_	Similar effects;
						Lower incidence of adverse reactions
	113 Liu, (2020)	TGT	Aza	Pred	_	No significant differences in autoantibodies, renal function changes, recurrence rate, and renal hemodynamic indexes
CAN	172 Ao et al. (1994)	TGT	Aza	CsA + Pred	_	Shorter times for renal function to return to normal after operation;
						Higher survival rate of transplanted kidneys in one to 2 years;
						Smaller risk of postoperative infection
	68 Tian and Liu (2020)	TGT	CTX	Sir + MMF + Pred + Val	—	Urine: 24 hUTP↓, α1-MG↓, β2-MG↓
	80 Wang et al. (2015b)	TGT	Usual care	CsA + Pred + MMF	_	Lower incidence of early acute rejection;
						Shorter times for creatinine to return to normal
	33 Li et al. (2008)	CRT	Usual care	CsA + Pred + MMF	60.0/22.0	Urine: 24hUTP↓
						Renal function: Scr↓, Ccr↑
						Others: better kidney function protection in the short-term

Abbreviations: Olm, olmesartan; Irb, irbesartan; FK506, tacrolimus; SBG, sex hormone binding globulin; E2, estradiol; T, testosterone; ARB, angiotensin II receptor blocker; PLA2R, phospholipase A2 receptor; Pred, prednisone or prednisolone; SF, sodium ferulate; Hyd, hydrochlorothiazide; Dip, dipyridamole; Lef, leflunomide; Dex, dexamethasone; IFN, interferon; Tel, telmisartan; GFR, glomerular filtration rate; Dapa, dapagliflozin; Val, valsartan; UACR, urine albumin creatine ratio; LDL-C, low-Density Lipoprotein Cholesterol; HDL-C, high-density lipoprotein cholesterol; SBP, systolic blood pressure; HGF, hepatocyte growth factor; Aza, azathioprine; CsA, cyclosporine A; Sir, sirolimus; MG, microglobulin; MMF, mycophenolate mofetil; Ccr, creatinine clearance rate.

TwHF preparations can also be applied to CAN, the chronic kidney injury after transplantation, which is an important factor affecting the effectiveness of kidney transplantation. CAN is a complex multi-causal process. Chronic rejection, recurrence of original kidney disease, newly acquired conditions and toxicity of immunosuppressive agents can all result in graft loss (Kielar et al., 2021). The pathology of kidney biopsy can indicate non-specific features such as chronic immune damage, interstitial fibrosis or renal tubular atrophy (Sayin et al., 2017). With the advancement of matching technology, surgical technology and the use of immunosuppressive agents, short-term outcome of kidney transplantation has been improved, but long-term outcome still remains a challenge (Gokhale et al., 2021). How to reduce the occurrence of CAN is currently the most concerned issue. New immunosuppressive agents are generally expensive and can cause serious side effects (Zhang et al., 2015). TwHF, as an immunosuppressant in Chinese medicine, has relatively small side effects and multiple targets due to its complex ingredients, which is consistent with the multi-factorial pathogenic characteristics of CAN (Zhang et al., 2015). A study had shown that although the immunosuppressive effect of tripterygium glycosides on kidney transplanted mice was not as good as cyclosporin A, it had a synergistic effect with cyclosporin A (Min, 1999). Therefore, the combination of TwHF preparations with immunosuppressive agents may increase the immune suppression effect and bring excess benefits such as renal function protection and side effects reduction according to table 4. The conclusion needs to be supported by large-scale clinical data.

### Mechanisms

**FIGURE 4:**
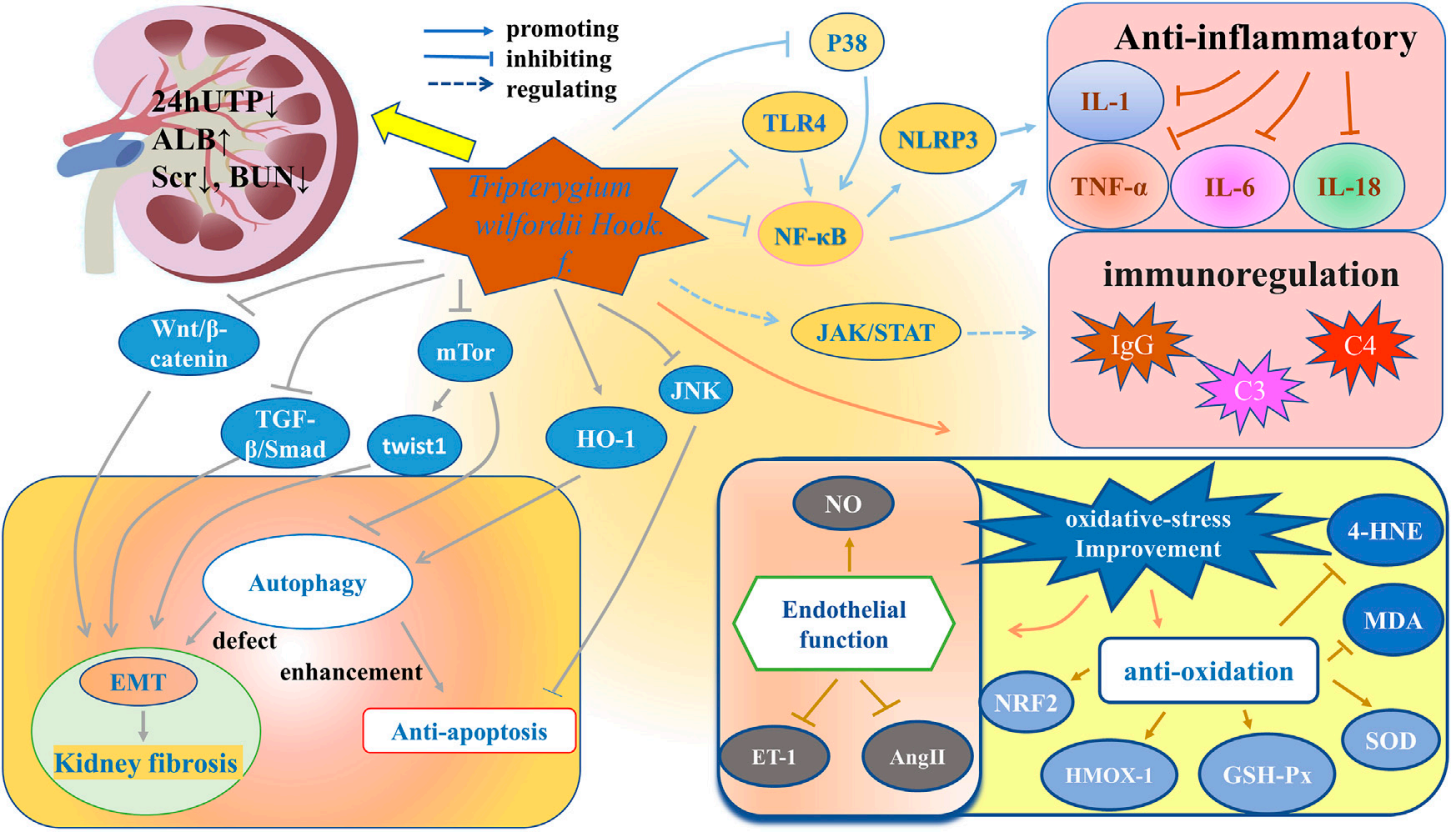
Targets and signaling pathways of TwHF and its preparations in treating nephropathy.

Anti-inflammatory and immune regulation are the main pharmacological effects of TwHF. In addition, TwHF has other pharmacological effects. For instance, Qin studied the effect of TP on podocyte pathology in Heymann nephritis model. He found that together with the improvement in the epithelial immune complex deposition, spikes, and basement membrane reactive proliferation, the dense on the epithelial side existed all along, even if proteinuria was significantly reduced (Qin et al., 2007). That is to say, besides the classic anti-inflammatory and immune regulation function, TwHF also has some other mechanisms to repair damage. Figure 4 generalizes the targets and signaling pathways of TwHF and its preparations in treating nephropathy.

#### Anti-inflammatory and Regulating Immunity

Inflammation and immunity are crucial pathogenesis of kidney diseases (Zhang and Sun, 2015). By stimulating human proximal tubular epithelial cells with inflammatory factors including IFN-γ (200 μg/L) and TNF-α (20 μg/L) for 24 h, Li et al. (2001) found that the expressions of major histocompatibility complex-Ⅱ (MHC-Ⅱ), intercellular adhesion molecule-1 (ICAM-1), and costimulatory molecules B7-1 and B7-2 were significantly increased. However, when TP was added in addition to IFN-γ and TNF-α at concentrations of 0.4 μg/L, 2.0 μg/L and 10.0 μg/L for 24 h, they found that the expressions of MHC-II and B7 decreased in a dose-dependent manner. The experimental results showed that TP weakened the ability of human proximal tubular epithelial cells to activate T cells as antigen-presenting cells and inhibited the inflammatory immune response (Li et al., 2001).

Nuclear factor- (NF-) κB signaling is a main signal pathway regulating inflammation and immunity (Morgan and Liu, 2011), which can be divided into classic and alternative pathways. The classic pathway is related to the response to pro-inflammatory cytokines-TNF-α and IL-1, involving the degradation of IκB protein. The alternative pathway does not involve the degradation of IκB, and plays an important role in immune organogenesis and lymphocyte function (Lawrence, 2009). It has been proven that in IgAN patients, NF-κB-mediated monocyte chemoattractant protein-1, granulocyte-macrophage colony stimulating factor and ICAM-1 are up-regulated in varying degrees in glomerulus and interstitium (Ashizawa et al., 2003). In LN and non-proliferative proteinuric glomerulopathy, podocyte NF-κB overactivation paralleled podocyte expression of TNF-α and IL-1β (Zheng et al., 2006). Based on the above findings about NF-κB signaling, a network pharmacology study of Liu et al. (2022) showed that the phosphatidylinositol 3 kinase (PI3K)/protein kinase B (Akt)/NF-κB/TNF-α/IL-1β pathway was the molecular mechanism of KXC to interfere DKD, which was confirmed by *in vitro* and *in vivo* experiments. Specifically, Liu et al. (2021) used high-fat diet combined with intraperitoneal injection of 35 mg kg^−1^ streptozotocin (STZ) to make DKD model in Sprague-Dawley (SD) rats. The KXC group was treated by KXC at a dosage of 480 mg kg^−1^, equivalent to 3 times of the clinical dosage for 4 weeks. The experimental results showed that compared with the control group without any treatment, the KXC treatment group can not only improve blood glucose, blood lipids, and urinary protein levels, but also significantly reduce phosphorylation-PI3K/PI3K, phosphorylation-AKT/AKT, phosphorylation-NF-κB/NF-κB, TNF-α and IL-1β levels (*p* < 0.01). The same results were also observed *in vitro* test (Liu et al., 2021).

Toll-like receptor 4 (TLR4) is the receptor of lipopolysaccharide, which is the outer wall component of gram-negative bacteria. TLR4 can activate the Toll/IL-1 receptor (TIR) and then activate the classic NF-κB pathway *via* adaptor protein with the TIR domain (Kawai and Akira, 2007; Zinatizadeh et al., 2021). Ma et al. (2015) also used high-fat diet combined with STZ to make diabetic model in SD rats. The diabetic rats were then randomly divided into four groups: diabetic rats without drug treatment and diabetic rats treated with TGT at a low-dose group (1 mg/kg/d), medium-dose group (3 mg/kg/d), and high-dose group (6 mg/kg/d), respectively. The administration time was 8 weeks. Through the detection of factors such as TLR4, NF-κB, and α-smooth muscle actin, they speculated that TGT can ameliorate renal tubulointerstitial fibrosis in diabetic rats through the TLR4/NF-κB signaling pathway (Ma et al., 2015).

The nucleotide-binding oligomerization domain-like receptor family pyrin domain-containing protein 3 (NLRP3), which expresses predominantly in macrophages, is also closely related to inflammation. It binds to the adaptor molecule ASC and controls the activation of caspase-1, and then caspase-1 cleaves pro-IL-1β and pro-IL-18. The activation process of NLRP3 inflammasome is also regulated by NF-κB signaling (Bauernfeind et al., 2009; Islam et al., 2020). A study showed that TP, intragastrically administered at a dosage of 200 mg/kg/d for 16 weeks, can down-regulate the expression of NLRP3 and TLR4 in rats with IgAN, thereby reducing serum IL-1β and IL-18 (He et al., 2015).

P38 is a member of mitogen-activated protein kinase (MAPK) family, and p38MAPK is another important pathway that mediates inflammation (Awasthi et al., 2021). The activation of MAPK can also activate NF-κB signaling pathway (Zhou et al., 2006). Zhang et al. (2019) evaluated the effect and influential mechanisms of celastrol on renal injury in diabetic rats. They established diabetic rat model with the intraperitoneal injection of STZ. Based on the administration dosage of celastrol, the celastrol group was divided into the low-dose group (50 μg/kg, 4 weeks) and the high-dose group (100 μg/kg, 4 weeks). The results showed that compared with the control group without the celastrol treatment, the celastrol group facilitates the protection of renal function, the improvement of renal histological lesions and the reduction of inflammatory factors. In addition, by comparing the levels of p38MAPK and NF-κB p65 with the MAPK/NF-κB inhibitor group, they proved that the function of celastrol can be attributed to the regulation of the MAPK/NF-κB pathway (Zhang et al., 2019).

Nephropathy is closely related to the disorder of immune system. IgAN, MN, focal segmental glomerulosclerosis and LN are all related to immune dysfunction (Kant et al., 2021). In an *in vitro* study of Hong et al. (2002), TNF-α (2.5 ng/ml)-induced human proximal tubular epithelial cells were exposed to TP, CsA, or FK506 for 24 h, and then the levels of C3, CD40, and B7h were tested. They found that TP at the concentration of 4–8 ng/ml can inhibit the expression of C3, CD40 and B7h, and it is even more effective than CsA and FK506 in inhibiting C3 (Hong et al., 2002). Zhang et al. (2017) treated MRL/lpr mice with LLDT-8 (0.125 mg/kg/2 days) for 9 weeks and found that LLDT-8 can reduce the levels of inflammatory factors such as IL-6, inhibit the expressions of chemokines such as IP-10 and the infiltration of renal T cells, and reduce the proportion of macrophages and neutrophils in MRL/lpr mice.

Janus kinase (JAK)/signal transducers and activators of transcription (STAT) pathway maintains the normal immune function. LN is a classic immune complex deposition disease (Alunno et al., 2019). It has been proven that JAK2-STAT1 inhibitor AG-490 can reduce the expression of IgG and C3, and reduce the inflammation of kidneys in LN rats, which suggests that the up-regulated JAK/STAT pathway is related to LN (Wang et al., 2010). Tang et al. (2021) intervened MRL/lpr mice with tail vein injection of TP (100 μg/kg) and found that the levels of JAK, p-JAK1, STAT3, and p-STAT3 decreased, while the levels of C3 and C4 and the positive rate of regulatory cells increased, which indicates that TP can reduce immune inflammatory response by inhibiting the JAK/STAT pathway.

#### Improvement of Oxidative Stress

Oxidative stress, inflammation and autophagy are closely related. Oxidative stress can increase the expression of pro-inflammatory IL-6, ICAM-1 and NF-κB (Prabhakar, 2013). Inflammation can induce oxidative stress, and oxidative stress can also up-regulate autophagy (Sureshbabu et al., 2015). Reactive oxygen species (ROS) can be divided into free radicals and non-free radicals. Although they play an important role in signal transduction, they have a strong binding capacity that can cause injuries to cellular components, such as lipids, proteins, and DNA (Tejchman et al., 2021). Oxidative stress is an important factor in inducing the progression of kidney diseases (Coppolino et al., 2018). It has been proven that oxidative stress can damage kidneys *via* increasing the synthesis of NADPH oxidases (Nlandu Khodo et al., 2012), regulating kelch-like ECH-associated protein1- (KEAP1-) nuclear factor erythroid 2-related factor 2 (NRF2) (Nezu et al., 2017), promoting endothelial mesenchymal (EnMT) (Li S. et al., 2021) and disrupting the balance with autophagy (Sureshbabu et al., 2015). Podocyte injury (Nagata, 2016), renal tubular fibrosis (Duni et al., 2019), CKD (Coppolino et al., 2018) and acute kidney injury (Sureshbabu et al., 2015) are all related to oxidative stress.

NO is a vasodilator, which can react with superoxide anion (O_2_^−^) to generate a strong oxidant: peroxynitrite (ONOO^−^), making NO relatively deficient. The lack of NO can trigger hypertension and subsequent proteinuria and glomerulosclerosis (Modlinger et al., 2004). Previous *in vivo* study showed that TGT can up-regulate the level of NO, down-regulate the levels of serum endothelin-1 (ET-1) and angiotensin-Ⅱ (AngⅡ), and lower TNF-α, IL-6, transforming growth factor- (TGF-) β1 levels to improve the vascular endothelial injury of CKD rats (Xu et al., 2021). Song et al. (2020) used tripterygium glycosides (50 mg/kg/d) to intervene DKD rats for 12 weeks and found that tripterygium glycosides can inhibit oxidation such as the decreased expression of malondialdehyde (MDA), and enhance the activity of antioxidant molecules such as the increased expression of superoxide dismutase (SOD), glutathione peroxidase (GSH-Px) and NRF2. 4-Hydroxynonenal (4-HNE) is the production of lipid peroxidation (Gao et al., 2010). Gao et al. (2010) found that both low-dose (25 μg/kg/d) and high-dose (50 μg/kg/d) TP for 12 weeks can reduce the expression of 4-HNE in db/db diabetic mice, especially for the high-dose group.

Oxidative stress is also related to vascular calcification, which is very common in patients with CKD (Li et al., 2019). Yang et al. (2021) first demonstrated that celastrol can inhibit vascular calcification from isolated vascular smooth muscle cells in SD rats, rat aortic rings and human arterial rings, respectively. Then they established CKD rat model by 5/6 nephrectomy. Alizarin red staining and Micro-CT analysis showed reduced aortic calcification in CKD rats treated with celastrol. Further, they found that vascular smooth muscle cells treated with celastrol significantly down-regulated ROS and MDA levels, while up-regulated the heme oxygenase-1 (HMOX-1) level. In addition, treatment with HMOX-1 inhibitor and HMOX-1 siRNA antagonized and blocked the effect of celastrol on vascular calcification. The results were verified in Vitamin D3-induced aortic calcification in mice. Based on the above findings, they concluded that celastrol attenuates oxidative stress and vascular calcification in CKD by upregulating HMOX-1 (Yang et al., 2021).

#### Enhancement of Autophagy and Anti-apoptosis

Autophagy is a cytoprotective mechanism, responsible for the degradation of long-lived or damaged proteins and dysfunctional organelles (Mariño et al., 2010). The maintenance of kidney structure and function relies on normal autophagy, the defect of which can lead to acute kidney injury and various primary or secondary nephropathy, and eventually result in fibrosis (Tang C. et al., 2020). Mammalian target of rapamycin (mTOR) is an important negative regulator of autophagy and is one of the downstream target proteins of Akt signaling pathway. An *in vivo* study conducted by Li et al. (2017) showed that 12 weeks of TP (200 μg/kg/d) treatment can up-regulate autophagy *via* miR-141–3p/PTEN/Akt/mTOR pathway to alleviate diabetic renal fibrosis.

Podocytes are a fundamental part of glomerular filtration membrane, the damage of which can directly lead to various kidney diseases (Afsar et al., 2021). Under external stimuli, podocytes can respond through hypertrophy, epithelial-mesenchymal transition (EMT) and apoptosis (Liu, 2010). Autophagy defects can trigger EMT (Li et al., 2015). Although the specific mechanism has not been clarified, EMT plays a critical role in interstitial fibrosis (Sheng and Zhuang, 2020). As a process of dedifferentiation, EMT is manifested by the disappearance of epithelial characteristics, such as the decreased or disappeared expression of nephrin, P-cadherin and zonula occludens-1, and the appearance of mesenchymal characteristics, such as the up-regulated expression of fibroblast-specific protein-1 and α-smooth muscle actin (Zhang et al., 2011). Tao et al. (2021) found that DKD mouse serum-induced podocytes *in vitro* decreased autophagy, and increased EMT and the expression of Twist1. When silencing Twist1, the results were reversed, so they believed that Twist1 was an important molecule that reduced autophagy and induced mesenchymal expression. Then, they found that TGT (1.25 μg/ml) was able to reduce Twist1 expression. However, after adding 3BDO, a mTOR activator, the expression of Twist1 increased, and the therapeutic effect of TGT was suppressed. Therefore, they concluded that TGT can suppress EMT in podocytes of DKD mice by targeting autophagy through the mTOR/Twist1 pathway (Tao et al., 2021).

In CKD, three signaling pathways (i.e., TGF-β/Smad, integrin/integrin-linked kinase (ILK), and Wnt/β-catenin) have been shown to be essential for tubular and podocyte EMT (Liu, 2010). Based on the above findings, a clinical study of Shen et al. (2021) found that TGT (20 mg, 3 times a day) combined with Pred for 12 months can reduce urinary TGF-β1 and Smad2 levels in patients with IgAN. Chang et al. (2007) found that the TGF-β1/Smad3 pathway is activated in rats with mesangial proliferative glomerulonephritis, and CRT (equivalent to clinical dosage) for 14 days can suppress the expression of TGF-β1 and Smad3. In addition, Chang et al. (2018) found that after 8 weeks of intervention in diabetic rats with 8 mg/kg and 16 mg/kg dosage of TGT, levels of Wnt-1, β-catenin, NF-κB p65 and TGF-β1 mRNA and protein were significantly reduced, so they speculated that the Wnt/β-catenin was involved in the effect of TGT on renal injury in diabetic rats, effectively preventing matrix accumulation and glomerulosclerosis.

Autophagy and apoptosis are two essential catabolic processes that maintain cell homeostasis and function. Despite significant differences between the two pathways, they are highly correlated in determining cell fate (Wu et al., 2014). Heme oxygenase-1 (HO-1) was proven to prevent high glucose-induced podocyte apoptosis *via* autophagy (Dong et al., 2015). Under high glucose condition, HO-1 is inactivated. Celastrol can restore the activity of HO-1 *in vitro* to protect podocytes from apoptosis, which is considered to be a new strategy for the treatment of DKD (Zhan et al., 2018). There are more findings about the anti-apoptotic effect of TwHF. For example, TP at the concentration of 2–4 mg/L can reverse the increased apoptosis rate of podocytes caused by PM2.5 (200 mg/L), which is manifested by the up-regulated protein expression of Bcl-2, nephrin and podocin, and the down-regulated protein expression of Bax (Wan et al., 2020). In terms of apoptosis, TP has also been shown to protect renal tubular epithelial cells from apoptosis caused by ischemia-reperfusion *via* c-Jun N-terminal kinase (JNK) signaling cascade reaction (Bao et al., 2018).

## Toxicity of TwHF Preparations

TwHF also called “Gelsemium *elegans*”. Leigong is the god of thunder in Chinese mythology. Thus, it can be seen that Chinese ancestors have long recognized the virulent toxicity of TwHF (Ren et al., 2021). The molecular basis of TwHF toxicity are diterpenoids, alkaloids, triterpenes and glycosides. Besides, TwHF also contains a relatively high amount of harmful elements, such as lead, arsenic and cadmium (Xue et al., 2010). According to statistics, the incidence of adverse reactions of TwHF preparations was 26.7% (Zhang C. et al., 2016). The adverse reactions included gastrointestinal toxicity (e.g., stomach pain, nausea, vomiting, diarrhea and anorexia), reproductive toxicity (e.g., decreased sperm count, menstrual disorders and amenorrhea), liver toxicity (e.g., jaundice and abnormal liver function), nephrotoxicity (e.g., hematuria, edema and oliguria), hematopoietic system toxicity (e.g., leukopenia), skin toxicity (e.g., scall), etc., among which gastrointestinal toxicity is the most commonly observed, followed by reproductive toxicity (Huang et al., 2016; Ru et al., 2019). Ding et al. (2021) found that 48 components and 78 key targets of TwHF were related to reproductive toxicity, which was mainly caused through regulating vasoconstriction. It is important to note that the therapeutic dosage and the toxic dosage of TwHF are very similar, that is to say, the efficacy and toxicity coexist, which aggravates the occurrence of adverse reactions (Liu LL. et al., 2019). A study showed that TwHF decoction in the range of 0.29–1.17 g/kg (equivalent to 4.5–18.2 times the clinical dose of 70 kg adults) has anti-inflammatory effects in mice and shows a dose-effect relationship. However, at the drug concentration of 0.585 g/kg, adverse reactions of liver toxicity have already occurred, and after reaching 2.34 g/kg, liver parenchyma damage occurs (Feng et al., 2014). In addition, Xu et al. (2001) found that for most of the people, by taking TGT at the dosage of 60 mg/day, the average cumulative dosage of inappetence, nausea, abdominal pain, and diarrhea were 7632 mg, 3780 mg, 3150 mg, and 6,750 mg, respectively; the average cumulative dosage of menstrual irregularity and menopause were 4,340 mg and 7710 mg, respectively; the average cumulative dosage of liver damage and rash were 15310 and 8,029 mg, respectively.

The toxicity of TwHF preparations is also related to dosage forms, pharmaceutical factories and whether they are combined with other drugs. For example, TwHF tablet, TwHF total terpenoids tablet and TwHF double-deck tablet are supplied by exclusive manufacturers, so their product contents and qualities are relatively stable. In contrast, TGT is currently supplied by 11 manufacturers^1^, and the current national drug standards for TGT do not include the quality standards of Leigongteng extract, so the contents and quality of the products produced by each manufacturer are different (Wang Y. D. et al., 2021), which increase the unsafety of clinical medication.

Therefore, for the use of TwHF preparations, special emphasis should be placed on the rationality of clinical medication (Kang et al., 2021), including grasping the medication population and indications, strictly following the usage and dosage, and closely monitoring the occurrence of adverse reactions. Moreover, strengthening drug quality supervision can also reduce the incidence of TwHF preparations’ adverse reactions. It is necessary to specify the drug standards of TwHF in different places of production, different seasons, and different parts when formulating drug standards to ensure the quality of the same preparation from different manufacturers (Sheng et al., 2017).

## Discussion

Our study introduces the development of main active components of TwHF and TwHF preparations and summarizes their applications and influential mechanisms in nephropathy. Considering the adverse reactions of TwHF and its preparations, we draw the following conclusions. First, the goal of “reducing toxicity and increasing efficiency” of TwHF preparations requires not only the discovery of new compounds and derivatives or the emergence of new dosage forms, but also the need to strengthen the quality management of existing preparations and the rational use of the drugs. Second, TwHF preparations combined with ARB or immunosuppressive agents may be better in reducing urine protein, protecting kidney function, reducing the incidence of adverse reactions, and maintaining long-term effects, which requires further verification by clinical large data. Third, TwHF treats nephropathy and protects kidneys mainly through anti-inflammatory and immune regulations, improving oxidative stress, and enhancing autophagy and anti-apoptosis.

Our research also has some limitations. For example, researches about the application of TwHF preparations in nephropathy is relatively limited. Most of the data are concentrated in China and focus on certain types of kidney diseases, so the application and mechanism of TwHF in nephropathy may not be comprehensive. As the “Chinese medicinal hormone”, TwHF has many components, which is quite likely to become a potential drug for the treatment of certain diseases in the future. It would be interesting to conduct more studies about the application of TwHF and its preparations in nephropathy and other fields.

## Footnotes

1 The data comes from National Pharmaceuticals Supervision Administration, web link: https://www.nmpa.gov.cn/datasearch/search-result.html.
